# Characterization of *CaHsp70-1*, a Pepper Heat-Shock Protein Gene in Response to Heat Stress and Some Regulation Exogenous Substances in *Capsicum annuum* L.

**DOI:** 10.3390/ijms151119741

**Published:** 2014-10-29

**Authors:** Meng Guo, Yu-Fei Zhai, Jin-Ping Lu, Lin Chai, Wei-Guo Chai, Zhen-Hui Gong, Ming-Hui Lu

**Affiliations:** 1 College of Horticulture, Northwest A&F University, Yangling 712100, China; E-Mails: guomeng19881025@126.com (M.G.); 18829785690@163.com (Y.-F.Z.); lujinpingok@163.com (J.-P.L.); 18717360273@163.com (L.C.); 2 Institute of Vegetables, Hangzhou Academy of Agricultural Sciences, Hangzhou 310024, China; E-Mail: kuni@21cn.com

**Keywords:** *Capsicum annuum* L., *CaHsp70-1*, heat stress, gene expression

## Abstract

Pepper (*Capsicum annuum* L.) is sensitive to heat stress (HS). Heat shock proteins 70 (Hsp70s) play a crucial role in protecting plant cells against HS and control varies characters in different plants. However, *CaHsp70-1* gene was not well characterized in pepper. In this study, *CaHsp70-1* was cloned from the pepper thermotolerant line R9, which encoded a protein of 652 amino acids, with a molecular weight of 71.54 kDa and an isoelectric point of 5.20. *CaHsp70-1* belongs to the cytosolic Hsp70 subgroup, and best matched with tomato *SlHsp70. CaHsp70-1* was highly induced in root, stem, leaf and flower in R9 with HS treatment (40 °C for 2 h). In both thermosensitive line B6 and thermotolerant line R9, *CaHsp70-1* significantly increased after 0.5 h of HS (40 °C), and maintained in a higher level after 4 h HS. The expression of *CaHsp70-1* induced by CaCl_2_, H_2_O_2_ and putrescine (Put) under HS were difference between B6 and R9 lines. The different expression patterns may be related to the differences in promoters of *CaHsp70-1* from the two lines. These results suggest that *CaHsp70-1* as a member of cytosolic *Hsp70* subgroup, may be involved in HS defense response via a signal transduction pathway contained Ca^2+^, H_2_O_2_ and Put.

## 1. Introduction

Pepper (*Capsicum annuum* L.), a member of the Solanaceae family, is a very important economic crop and has a wide variety of uses, including as a consumable vegetable, a condiment in cuisines, as pharmaceuticals and as coloring agents [1,2]. Pepper is a typical non-thermophyte, during its life cycle, it is always affected by stresses such as extreme temperature, especially heat stress (HS), which can cause devastating yield and quality loses [3,4]. For instance, HS inhibited the pepper seed vigor, reduced chlorophyll content of leaves that resulted in lower photosynthesis rate, induced increases in total, cytochrome and alternative pathway respirations, resulted in fertilization failure, and eventually cause flower and fruit dropping [5,6].

Under HS condition, the synthesis of most normal proteins in organisms was inhibited, while heat shock proteins (Hsps) were rapidly synthesized. As a key kind of molecular chaperones, Hsps functioned in mitigating the effects of HS on plants and were considered to be responsible for the acquisition of thermotolerance [7]. In higher plants, based on the approximate molecular weight, Hsps are classified into five major families: small heat shock protein (sHsp), heat shock protein 60 (Hsp60), Hsp70, Hsp90, and Hsp100 [8]. Among these Hsps, Hsp70 family is the most to focus on, is fundamental in plant developmental processes and function in HS. In *Arabidopsis*, the heat-inducible *Hsp70s* in mature dry seed and roots played prominent roles in seed maturation and root growth [9]. The *Arabidopsis* stromal *cpHsc70-1* mutant plants exhibited variegated cotyledons, malformed leaves, growth retardation, and root growth was impaired seriously after treating germinating seeds with HS [10]. Hsp70s also have been reported to play roles in responses to HS in *Hevea brasiliensis* [11], *Oryza sativa* [12], *Nicotiana tabacum* [13], and *Triticum aestivum* [14].

The function of Hsp70s is based on their structures. Hsp70 family which comprised two major functional domains is recognized as the most conserved Hsps families. In this family, an approximately 40-kDa *N*-terminal adenosine triphosphatase (ATPase) domain (also called nucleotide binding domain; NBD) and a 25-kDa *C*-terminal peptide-binding domain, are separated by a hinge region [8,15]. In plant, Hsp70s are categorized into four major subgroups based on the unique and highly conserved *C*-terminus motif, including the cytosolic, endoplasmic reticulum (ER), mitochondrion (MT) and chloroplast (CP) group [15,16,17]. Due to their location in different subcellular compartments, the Hsp70 proteins may play specific roles at different stages of development or interact with specific associated proteins [8,18]. Despite Hsp70 is a well characterized family, there are limited functional studies of plant cytosolic Hsp70s [19].

For pepper, the roles of various genes, such as *CaWRKY40* [20], *CaBI-1* [21], *CaRZFP1* [22] under HS have been reported. However, investigations of the molecular basis of heat tolerance in pepper at the Hsp level are poor. In this respect, only few members, *CaHsfA2* [4], *CaHsp24* [23] and *CaHsp22.5* [24] have been reported as responding to HS. Although Hsp70s play a key role in enhancing the heat resistance of plants, the function of Hsp70s in the process of pepper resistance to HS rarely has been reported.

In this study, an *Hsp70* gene was cloned from thermotolerant pepper line R9 named *CaHsp70-1* (GenBank accession No. KC176708) by rapid amplification of cDNA ends (RACE) technique, and analyzed the deduced protein sequence with a series of bioinformatic tools. In addition, we studied the subcelluar localization of CaHsp70-1 and investigated the expression patterns of *CaHsp70-1* in pepper under HS and some regulation exogenous substances, also cloned the promoters of *CaHsp70-1* from both thermosensitive line B6 and thermotolerant line R9, and compared the difference between the *cis*-elements from the two promoters.

## 2. Results and Discussion

### 2.1. Isolation and Sequence Analysis of CaHsp70-1

**Figure 1:**
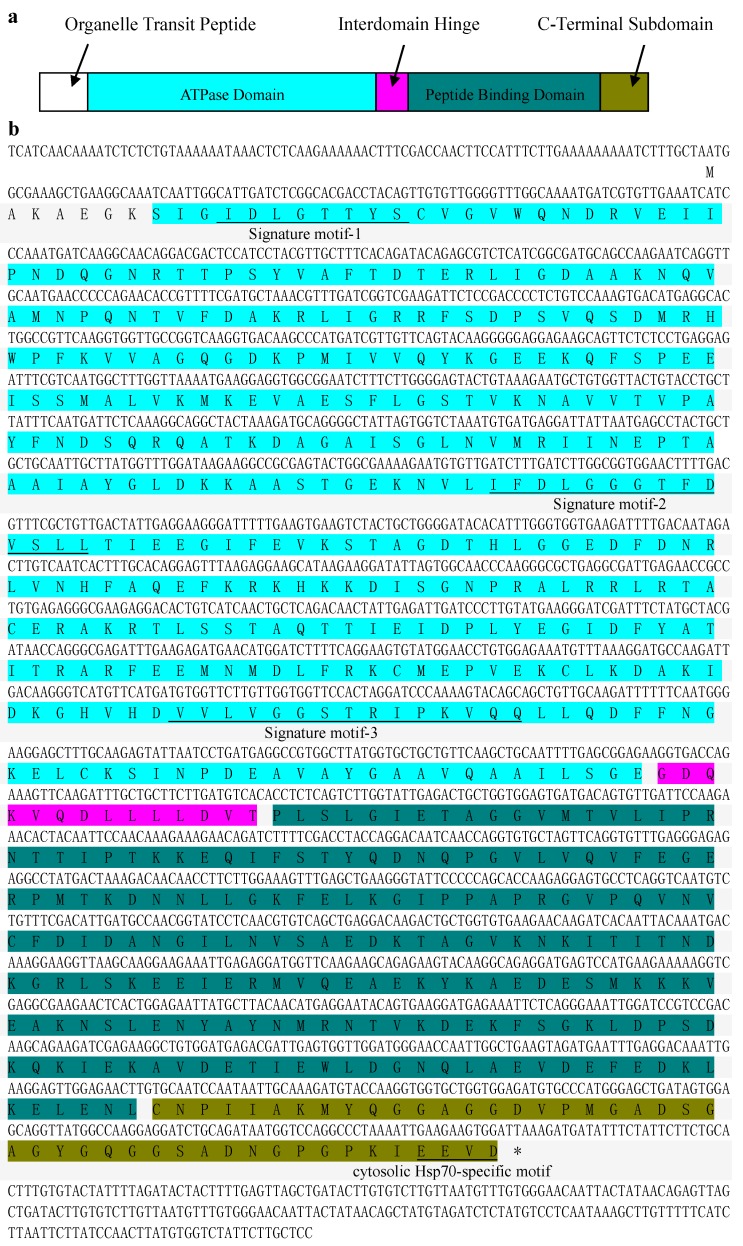
Nucleotide sequence and deduced amino acid sequence of *CaHsp70-1*. (**a**) Schematic representation of CaHsp70-1 protein structure; (**b**) The nucleotide sequence and amino acid sequence of CaHsp70-1. A putative open reading frame (ORF) started with ATG and ended with TAA. An asterisk below the last three nucleotides indicates a stop codon; *Hsp70* signature motifs and cytosolic *Hsp70*-specific motif are underlined. The sequence is color coded to match the diagram in (**a**).

Based on the affinity between tomato (*Solanum lycopersicum* L.) and pepper, the gene we identified on the basis of the sequence of the tomato heat shock cognate 70 kDa protein-like gene (*SlHsp70*; XP 004235887.1) was designated as *CaHsp70-1* (GenBank accession No. KC176708). The full length of *CaHsp70-1* cDNA was 2288 bp, with a 5' untranslated region (5' UTR) of 87 bp, a 3' untranslated region (3' UTR) of 242 bp and an ORF of 1959 bp, which encoded 652 amino acids (AA) with a molecular weight of 71.54 kDa and an isoelectric point of 5.20. The deduced protein sequence contained three signature motifs of the Hsp70 family, IDLGTTYS, IFDLGGGTFDVSLL, and VVLVGGSTRIPKVQQ, located at residues 11–18, 202–215, and 339–353, respectively (Figure 1b) [15,25]. In addition, CaHsp70-1 protein contained two functional domains of Hsp70 family at *C*-terminal, an *N*-terminal ATPase domain and a peptide binding domain, which linked by a hinge region (Figure 1). The *C*-terminus protein sequence was EEVD, which showed the highly conserved sequence of cytosolic Hsp70 subgroup.

**Figure 2:**
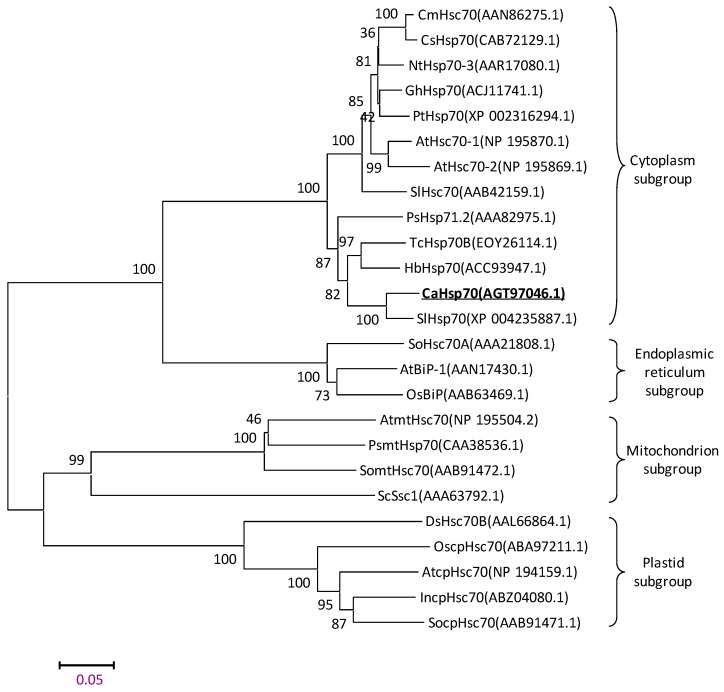
Neighbor-joining-based phylogenetic tree of heat shock protein 70 family members. The tree was computed using MEGA 5.1 program. Accession numbers of the Hsp70 sequences analyzed are AAN86275 (*Cucurbita maxima* CmHsc70), CAB72129 (*Cucumis sativus* CsHsp70), AAR17080 (*Nicotiana tabacum* NtHsp70-3), ACJ11741 (*Gossypium hirsutum* GhHsp70), XP_002316294 (*Populus trichocarpa* PtHsp70), NP_195870 (*Arabidopsis thaliana* AtHsc70-1), NP_195869 (AtHsc70-2), AAB42159 (*Solanum lycopersicum* LeHsc70), XP 004235887.1 (SlHsp70), AAA82975 (*Pisum sativum* PsHsp71.2), AGT97046 (*Capsicum annuum* CaHsp70-1), EOY26114 (*Theobroma cacao* TcHsp70B), ACC93947 (*Hevea brasiliensis* HbHsp70), AAA21808 (*Spinacia oleracea* SoHsc70A), AAB63469 (*Oryza sativa* OsBiP), AAN17430 (*Arabidopsis thaliana* AtBiP-1), NP_195504 (AtmtHsc70), CAA38536 (*Pisum sativum* PsmtHsp70), AAB91472 (*Spinacia oleracea* SomtHsc70), AAA63792 (*Saccharomyces cerevisiae* ScSsc1), AAL66864 (*Dunaliella salina* DsHsc70B), ABA97211(*Oryza sativa Japonica Group* OscpHsc70), NP_194159 (*Arabidopsis thaliana* AtcpHsc70), ABZ04080 (*Ipomoea nil* IncpHsc70), and AAB91471 (*Spinacia oleracea* SocpHsc70).

In order to confirm whether CaHsp70-1 belongs to cytosolic Hsp70 subgroup, Hsp70s from plants, yeast, and algae were collected for sequence alignment and constructing the neighbor-joining phylogenetic tree. As shown in Figure 2, in the phylogenetic tree of Hsp70s, CaHsp70-1 was clustered together with cytosolic Hsp70s, and BLAST analysis showed that the deduced amino acid (AA) sequence for CaHsp70-1 shared significant identity (94%) to SlHsp70. Furthermore, the prediction provided by WoLF PSORT showed a cytoplasm localization of CaHsp70-1 protein. It was worth noting that the proteins from the same subcellular compartments had closer relationships, such as *Spinacia oleracea* SoHsc70A (AAA21808), *Oryza sativa* OsBiP (AAB63469), and *Arabidopsis thaliana* AtBiP-1 (AAN17430) which were clustered into ER Hsp70 subgroup, and had higher homology than other subcellular Hsp70 families.

To check the intron insertion into *CaHsp70-1* gene, we cloned the full-length DNA and cDNA sequences with the full-length primers and the templates of DNA and cDNA from R9 leaves. The result showed that the amplification products of DNA and cDNA had the same length bands, indicating no intron in *CaHsp70-1* gene (not shown), which was accordant with the fact that genes encoding cytosolic Hsp70s usually have zero or one intron [15].

According to the current results of protein sequence analysis, phylogenetic tree of Hsp70s, prediction of subcellular localization and *CaHsp70-1* gene intron analysis, we suggested that CaHsp70-1 belonged to cytosolic Hsp70 family.

### 2.2. CaHsp70-1 Localizes to the Cytoplasm and the Nucleus

**Figure 3:**
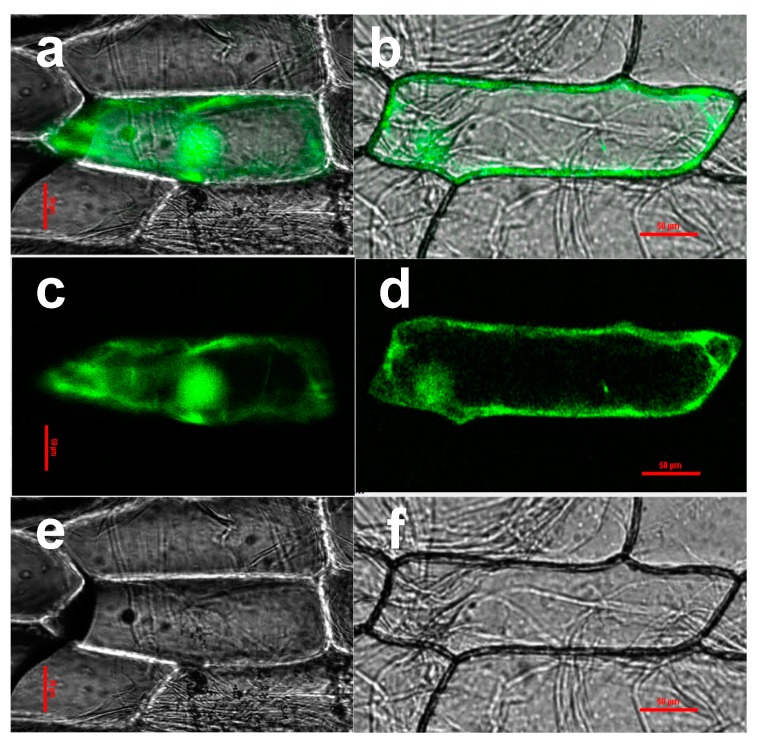
Transient expression of the CaHsp70-1–GFP fusion protein in onion epidermal cells. CaMV35S, a constitutive promoter from the cauliflower mosaic virus. GFP, green fluorescent protein; NOS, nopaline synthase terminator. Transient expression of GFP and CaHsp70-1–GFP in onion epidermal cells. (**a**,**c**,**e**) Onion epidermal cells transformed with 35S::GFP as control; (**b**,**d**,**f**) Onion epidermal cells transiently expressing 35S::CaHsp70-1–GFP; (**a**,**b**) Merged images; (**c**,**d**) Dark field images; (**e**,**f**) Bright field images. Bars = 0.5 mm.

The deduced protein sequence contains a cytosolic Hsp70-specific motif of EEVD at the end of the CaHsp70-1 *C*-terminus (Figure 1b), while it also contains sequences associated with nuclear targeting. To clarify whether CaHsp70-1 protein localizes to the cytoplasm or the nucleus, fusion proteins of CaHsp70-1 and green fluorescent protein (GFP) were used to determine the subcellular localization of CaHsp70-1, and expression of the fusion proteins was driven by the cauliflower mosaic virus CaMV35S promoter. Following particle bombardment into onion epidermal cells, CaMV35S::CaHsp70-1-GFP was expressed in the cytoplasm and the nucleus. While, the control GFP was uniformly distributed throughout the cell (Figure 3).

### 2.3. CaHsp70-1 Expression in Pepper Tissues and Its Responses to Heat Stress (HS) Treatment

**Figure 4:**
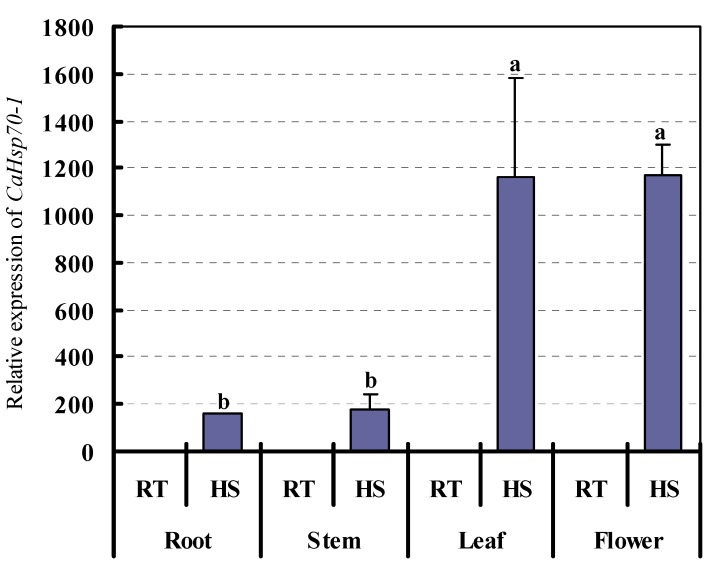
Expression of *CaHsp70-1* in R9 tissues. R9, pepper thermotolerant line; RT, room temperature (26 °C for 2 h); HS, heat stress (40 °C for 2 h). Expression data were normalized with *UBI-3* as reference gene. The expression levels are relative to that of the leaf from R9 under RT. Error bars indicate the mean and standard deviation (*n* = 3), and the different lowercase letters show the significant difference at α = 0.05 level. The following are the same.

In order to identify whether Hsp70 members involved in plant developmental processes and had function under HS or not, we investigated the *CaHsp70-1* expression pattern in different pepper tissues, including root, stem, leaf and flower from R9 plants. Figure 4 showed that although *CaHsp70-1* was present in all tissues, it was strongly induced in tissues which treated with 40 °C for 2 h, especially in leaves and flowers. In addition, the expression level in flowers exposed to HS was the highest compared with the corresponding untreated tissue.

**Figure 5:**
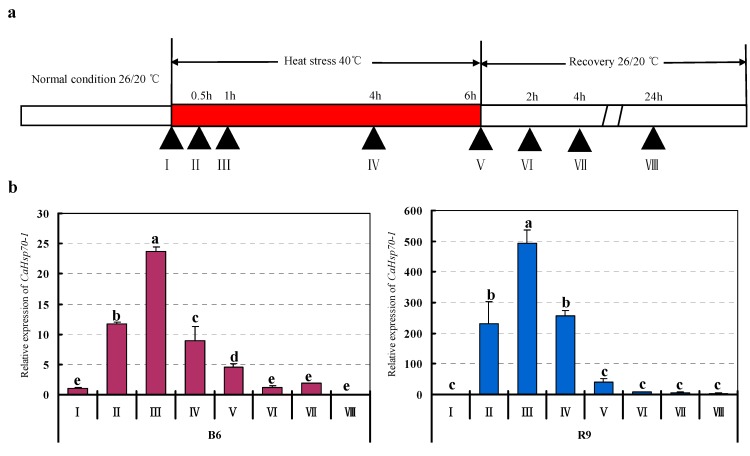
Expression of *CaHsp70-1* in pepper responses to HS treatment. B6, pepper thermosensitive line; R9, pepper thermotolerant line. (**a**) The time course of HS treatments. Triangles indicate the time points when the leaves were collected (sample I–VIII); (**b**) Expression profiles of *CaHsp70-1* in pepper leaves by HS treatment at different time points. Expression data were normalized with *UBI-3* as reference gene. The expression levels are relative to that of the sample I from B6 and R9, respectively.

For further analysis to the effect of HS on *CaHsp70-1* expression, HS experiment (with thermosensitive line B6 and thermotolerant line R9) was designed. The young leaves were collected at different times after starting of HS treatment and recovery treatment (Figure 5a). In both B6 and R9, the expression profiles of *CaHsp70-1* were almost identical, and the expression level of *CaHsp70-1* significantly increased after 40 °C for 0.5 h (Figure 5b, sample II), and still maintained a high level until 4 h (Figure 5b, sample IV). During the process of HS treatment, the induced expression level in R9 leaves was higher than B6. After the HS treatment for 6 h, the seedlings were removed to normal temperature condition, under which *CaHsp70-1* expression were barely detectable in leaves for a 24 h recovery (samples VIII in Figure 5b). *CaHsp70-1* was more intense response to HS in thermotolerant line R9 than in thermosensitive line B6 plants.

### 2.4. Effect of CaCl_2_, H_2_O_2_ and Put on the Expression of CaHsp70-1 in Pepper Leaves under HS

**Figure 6:**
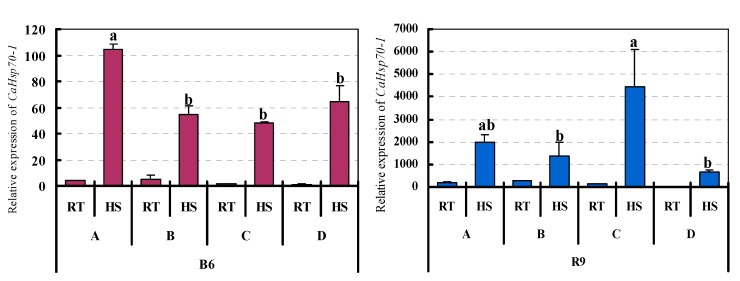
Expression of *CaHsp70-1* in pepper leaves treated with exogenous substances and HS. The pepper seedlings were sprayed with different exogenous substances or water once a day, continuous for three days, then implemented heat shock (40 °C) or room temperature (26 °C) for 2 h. (A) Treated with CaCl_2_ (15 mM); (B) Treated with H_2_O_2_ (1.0 mM); (C) Treated with Put (1.5 mM); (D) Treated with water. Parts of (A–D) seedlings were treated with HS, others were treated with room temperature. B6, pepper thermosensitive line; R9, pepper thermotolerant line; RT, room temperature (26 °C for 2 h); HS, heat stress (40 °C for 2 h). Expression data were normalized with *UBI-3* as reference gene. The expression levels are relative to that of the sample (D) with RT treatment in B6 and R9, respectively.

During HS, many substances, such as Ca^2+^, H_2_O_2_, and PAs, are involved in the signal transduction pathways for perceiving the heat and mediating the HS response [26,27,28,29]. *CaHsp70-1* was induced by CaCl_2_, H_2_O_2_ and Put compared with water (control) under room temperature (Figure 6). Under HS, *CaHsp70-1* expression in R9 was further induced by CaCl_2_, H_2_O_2_ and Put than under room temperature. Moreover, the transcription level induced byPut was higher than the other two exogenous substances. In B6, the expression levels of *CaHsp70-1* in treatments with these exogenous substances were lower than the control treated with water except for CaCl_2_ (Figure 6).

### 2.5. The Promoter of CaHsp70-1 Analysis

**Figure 7:**
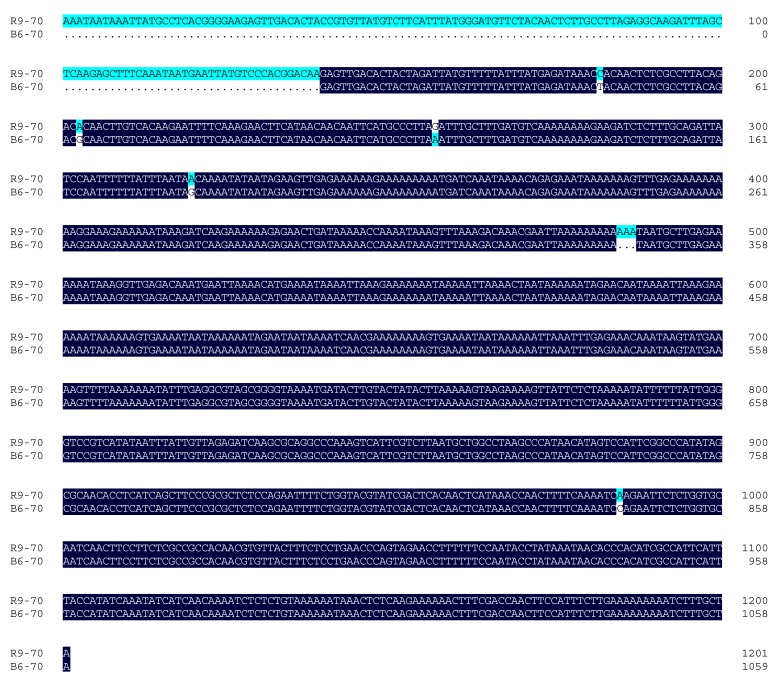
Alignment of promoter sequences of *CaHsp70-1* from B6 and R9. Dark color indicates the consensus sequences, and light color indicates the different sequences.

As the gene expression was regulated by transcription factors or effectors binding to its upstream promoter elements, we also analyzed the promoter regions of *CaHsp70-1* from B6 and R9 (Table 1). There were three HSEs and two TC-rich repeats elements (*cis*-acting element involved in defense and stress responsiveness) in the promoters of *CaHsp70-1* in both B6 and R9 cultivars. However, comparing of the promoters in R9 (1201 bp) and B6 (1059 bp), there were 142 bp absent in the promoter from B6 (Figure 7). This missed part in the promoter included one Box I (light responsive element), one CAAT-box (common *cis*-acting element in promoter and enhancer regions), two TATA-box (core promoter element around −30 of transcription start) and one Unnamed-1/Unnamed-3 element. In addition, the promoter of *CaHsp70-1* from B6 lacked one Unnamed-1/Unnamed-3 element compared with that of R9. While the ABRE element (*cis*-acting element involved in the abscisic acid responsiveness) was lacked in the promoter of *CaHsp70-1* from R9.

**Table 1:** *cis*-Elements in promoters of *CaHsp70-1* from B6 and R9.

Name of *cis*-Elements	Sequences	Number of *cis*-Elements		Function
		R9	B6	
AAGAA-motif	GAAAGAA	1	1	unknown
ABRE	GCAACGTGTC	0	1	*cis*-acting element involved in the abscisic acid responsiveness
AE-box	AGAAACAA	1	1	part of a module for light response
ARE	TGGTTT	2	2	*cis*-acting regulatory element essential for the anaerobic induction
AT1-motif	AATTATTTTTTATT	1	1	part of a light responsive module
Box I	TTTCAAA	3	2	light responsive element
Box II/III	ATCATTTTCACT	3	3	protein binding site
CAAT-box	CAATT/CCAAT/CAAT/CAAAT	17	16	common *cis*-acting element in promoter and enhancer regions
CGTCA-motif	CGTCA	1	1	*cis*-acting regulatory element involved in the MeJA-responsiveness
EIRE	TTCGACC	1	1	elicitor-responsive element
G-Box	CACGTT/TAACACGTAG	3	3	*cis*-acting regulatory element involved in light responsiveness
GARE-motif	AAACAGA	1	1	gibberellin-responsive element
HSE	CNNGAANNTTCNNG/AAAAAATTTC/AGAAAATTCG	3	3	*cis*-acting element involved in heat stress responsiveness
I-box	CCATATCCAAT	1	1	part of a light responsive element
SARE	TTCGACCATCTT	1	1	*cis*-acting element involved in salicylic acid responsiveness
Skn-1_motif	GTCAT	2	2	*cis*-acting regulatory element required for endosperm expression
TATA-box	TTTTA/TAATA/ATATAAT/TATA/TTTAAAAA/TCTATAAATA/CCTATAAAAA	42	40	core promoter element around −30 of transcription start
TC-rich repeats	GTTTTCTTAC/ATTCTCTAAC	2	2	*cis*-acting element involved in defense and stress responsiveness
TCT-motif	TCTTAC	1	1	part of a light responsive element
TGACG-motif	TGACG	1	1	*cis*-acting regulatory element involved in the MeJA-responsiveness
Unnamed__1/3	CGTGG	2	0	unknown
Unnamed__4	CTCC	2	2	unknown
chs-CMA2b	GTATCTACTCAC	1	1	part of a light responsive element
circadian	CAAAGATATC/CAANNNNATC	2	2	*cis*-acting regulatory element involved in circadian control
rbcS-CMA7a	GGCGATAAGG	1	1	part of a light responsive element

### 2.6. Discussion

The stress conditions always adversely affect the growth and development of pepper; for example, extreme temperature can disturb pollination and fertilization, resulting in a decline in production and quality of pepper fruits [1,3]. Although the essential function in plants of fighting against environmental stress has been studied in various species [9], few studies focus on the role of pepper *Hsp70s* in the stress-defense process. In this paper, we cloned a cytosolic heat-shock protein gene *CaHsp70-1*, analyzed the deduced protein sequence, and investigated the expression patterns under HS and treatments with exogenous substances.

Because of unique and highly conserved, the *C*-terminus of plant Hsp70s can be used to distinguish the protein’s subcellular location [15]. CaHsp70-1 protein sequence contained an EEVD protein sequence at *C*-terminus (Figure 1), which is associated with substrate specificity and can interact with specific co-chaperones in the Hsp70 family [30]. Based on the phylogenetic tree, *CaHsp70-1* was clustered into cytosolic subgroup, and best matched with *Hsp70* of *Solanum lycopersicum* (Figure 2), which agreed with their botanical classification. CaHsp70-1 was predicted as a cytoplasm localization, which ensured that it contained an EEVD protein sequence at *C*-terminus. Subcellular localization of CaHsp70-1 is shown in Figure 3, which confirmed that it localized to the cytoplasm and nucleus. Song *et al.* reported that CgHsp70 from chrysanthemum deposited in the cytoplasm and nucleus, although it contained the cytoplasm protein sequence EEVD at *C*-terminus [31]. Under heat shock stress, Hsp70s were rapidly relocated from the cytoplasm into the nucleus, *O*-GlcNAc glycosylation, cochaperone Hsp110, Hsp40 and the nuclear import carrier for Hsp70s Hikeshi involved this process by a yet unknown mechanism [32,33]. CaHsp70-1 located to the nucleus, which suggested that it might counteract the damage of cells from HS by acting inside the nucleus [33]. However, as for pepper cytosolic *CaHsp70-1*, its biological function still needed further research.

Many *Hsp70* genes involved in plant developmental stages are responsive to HS and correlated with the thermotolerance [9]. The expression level of *CaHsp70-1* under HS was higher than under room temperature condition. Furthermore, in room temperature condition, the expression level of *CaHsp70-1* in leaves (1.02) was higher than in the other three tissues (0.22–0.28). While under HS treatment, *CaHsp70-1* level was further induced in flowers than other three vegetative tissues (Figure 4). Previous studies reported that intensive protein synthesis occurs in the plant reproductive tissues [34], and plant Hsp70s involved in developmental regulation during the reproductive phase [15], but *CaHsp70-1* expression level was highest in leaves under normal room temperature consisted with the expression of *BccpHsc70-2* [35], which might be related to the stages of reproductive phase and/or the function of itself. In tomato *Hsc70* transcripts we detected in mature anthers, but not in pollen [34], while many other *Hsp70* genes were abundant during seed maturation and/or germination, but not detected during other developmental stages [9]. Tomato *HsfA2* and *Hsp17.4-CII* genes as the potential members during the HS response, their expression was more detectable during development at the 2 mm anthers stage under control temperature than during other different sized anthers (4, 6, or 8 mm) stages [36]. The tissue of pepper flowers were collected during the alabastrum stage and used for investigating the transcription level of *CaHsp70-1*, which might be not the high expression level stage during the anther development phase. Besides, with the help of cytosolic Hsp70s, precursor proteins synthesized in the cytoplasm were transferred from cytosolic Hsp70s to chloroplast Hsp70s [18]. These results suggested that *CaHsp70-1* played a specific role in chloroplasts abundant in leaves. *CaHsp70-1* level was further induced in flowers by HS which proved that *CaHsp70-1* might be involved in the renaturing of proteins under HS conditions, keeping the proteins conformation of flower organs normal, ensured the normal pollination and fertilization.

With the continuous HS treatment of 40 °C, the expression of *CaHsp70-1* from both cultivars was strongly and rapidly induced at 0.5 h for HS (sample II in Figure 5b). Once the plants suffered from HS, many Hsps, such as small Hsps, Hsp70s and Hsp100s were rapidly induced to prevent unfolded proteins from undergoing nonproductive aggregation, thus protecting plants from HS damage [19]. The rapid response of *Hsp70* genes to HS has been described in various plants [9,11,17], which suggested that many *Hsp70s* were associated with the HS tolerance. Many previous studies reported that higher *Hsp70* expression could increase heat tolerance [37,38]. In accordance with the previous results we observed that *CaHsp70-1* was also associated with pepper HS tolerance. However, not all *Hsp70s* were induced by HS, the expression of *cpHsc70-1* and *mtHsc70-1* from organellar members of the *Arabidopsis Hsp70* gene family were unchanged or repressed [9]. Although in wheat *TaHsc70* expression increased in leaves of etiolated seedlings after HS, remained stable in green seedlings [14]. It was noteworthy that the *CaHsp70-1* transcript levels induced by HS treatment (40 °C for 0.5, 1, 4, 6 h corresponding to sample II, III, IV and V, respectively in Figure 5b) in R9 leaves were always higher than that in B6 leaves. These results were in the same line with the level of *BccpHsc70-1* expression in the Non-Heading Chinese Cabbage thermotolerant and thermosensitive cultivars. Thus, it could be suggested that the level of *CaHsp70-1* accumulation was associated with the HS tolerance [31].

The response to HS is a complex signal transduction process in plants, in which many signal substances, such as Ca^2+^ and H_2_O_2_, are involved by producing a series of secondary metabolites and inducing or activating relative genes expression [39,40]. PAs have an important role in regulating plant growth and development, aging and response to stress [41], and Put, as one form of PA, can regulate cell pH and reactive oxygen metabolic balance and stabilize membrane structure under stress conditions [42]. CaHsp70-1 contained a CaM-binding site, a 21-amino acid sequence, PRALRRLRT ACERAKRTLSST at positions 262–282, showed 100% similarity with that from maize cytoplasmic Hsp70, which bound CaM in a Ca^2+^-dependent manner [43]. In the current results, under HS, *CaHsp70-1* expression in B6 and R9 was further induced by CaCl_2_. It is interesting to note that the expression level induced by the combination treatment (HS with CaCl_2_) was higher than plants treated with HS or CaCl_2_, also higher than the summation expression level of both HS and CaCl_2_ treatments(Figure 6). HS causes a rapid increase in intracellular free calcium [44,45], and Ca^2+^ is involved in the expression of *Hsps* [46,47]. Under HS conditions, the intracellular concentration of calcium ions increased, directly activated CaM, cytosolic CaHsp70-1 bound to CaM causing the Hsf–Hsp70 complex to release Hsfs which binds to HSE, and activated transcription of *Hsp* genes, including *CaHsp70-1* gene [43]. The combination treatment (HS with CaCl_2_) might have the synergistic effecting on *CaHsp70-1* induced expression. *CaHsp70-1* was induced by H_2_O_2_ and Put, but it had diverse expression patterns in B6 and R9. The transcript level was induced by the combination treatment (HS with H_2_O_2_, and Put) in R9, was lower than that treated with HS in B6, which suggested that the combination treatment (HS with H_2_O_2_, and Put) had the diverse effect on *CaHsp70-1* expression in both thermosensitive and thermotolerant lines (B6 and R9). The role of combination treatments in *Hsp* genes expression depended on the thermostability of the plants. This result indicated that in the complex signal transduction process, HS interacted with CaCl_2_, H_2_O_2_ and Put, co-regulated the *CaHsp70-1* expression.

The difference between B6 and R9 (treated with HS and exogenous substances) in the expression level of *CaHsp70-1* was obvious. Therefore, we speculated that the mechanism of differential *CaHsp70-1* expression in B6 and R9 was related to the regulation of promoter of *CaHsp70-1*. The induction of *Hsp70s* under HS condition is regulated by heat shock transcription factors (Hsfs) and corresponding heat shock elements (HSEs) in the promoters [15]. In the present results, we also cloned and analyzed the promoters of *CaHsp70-1* to confirm whether there were differences between the two different lines (one sensitive and the other is tolerant to heat stress) or not, which might be related to the expression patterns of *CaHsp70-1* (Figure 7; Table 1). Among these *cis*-elements, the HSE and TC-rich repeats elements in the promoters of *CaHsp70-1* from B6 and R9 might be involved in the response to HS and exogenous substances. However, there are several differences that exist in the promoters of *CaHsp70-1* from B6 and R9, and we speculated that differences might be the reason for different expression patterns and levels of *CaHsp70-1* from B6 and R9, which led to the spatial and temporal differences in *CaHsp70-1* expression in different cultures (Figure 5 and Figure 6)*.* To clarify the relationship between *CaHsp70-1* expression and exogenous substances in pepper plants under HS, the dynamic change of *CaHsp70-1* expression level and the function of promoter of *CaHsp70-1* in the process of exogenous substances and HS treatments need to be further studied.

## 3. Experimental Section

### 3.1. Materials, Growth Condition and Treatments

The pepper thermotolerant line R9 was introduced from Asia Vegetable Research and Development Center (AVRDC), and the thermosensitive line B6 was selected by pepper research group in College of Horticulture, Northwest A&F University (Yangling, China). Both lines were grown in a growth chamber at 26/20 °C day/night and a 16/8 h day/night photoperiod till reaching the age of 6–8 true leaves. For *CaHsp70-1* gene intron analysis, the R9 leaves were collected, frozen with liquid nitrogen and stored at −80 °C prior to extraction of DNA. For HS treatment, after being subjected to 40 °C for 0, 0.5, 1, 4, 6 h, and recovered at room temperature (26/20 °C day/night) for 2, 4, 24 h after HS treatment for 6 h, the leaves in B6 and R9 were collected, respectively (Figure 5a). For the exogenous substances treatment, seedlings were sprayed with CaCl_2_ (15 mM), Put (1.5 mM), H_2_O_2_ (1.0 mM) and water (control), once daily, for continuous three days. On the fourth day, these seedlings were treated with HS of 40 °C for 2 h by placing in a light incubator (GXZ-380C, Jiangnan Instrument Factory, Ningbo, China), and others under room temperature for 2 h were used as the control. After HS treatment, the treated young leaves (with different exogenous substances) were collected at the same time. At the time of flower buds appearance, R9 plants were used for the tissue specific expression analysis. The young leaves, flower buds, stems and roots of treated and untreated plants were collected and frozen with liquid nitrogen for the total RNA extraction and cDNA synthesis. Each treatment was conducted with three biological replicates, and samples from five seedlings were gathered for each replicate.

### 3.2. DNA and Total RNA Isolation, cDNA Synthesis

DNA of pepper leaves was extracted by CTAB method, and total RNA were isolated according to the instruction of Total RNA kit (BioTeke, Beijing, China). For reverse transcription, total RNA was used according to the manufacturer’s instructions (Takara, Dalian, China).

### 3.3. Cloning the CaHsp70-1 Gene

Total RNA was extracted from the young leaves in R9 which have been exposed to HS (40 °C for 2 h), and used to synthesize first strand of cDNA. The sequence of *CaHsp70-1* was partly obtained by homology-based candidate gene method from the published pepper expressed sequence tags, by which the *SlHsp70* (XP 004235887.1) sequence was used as the information probe. The sequences of the 5' and 3' regions of *CaHsp70-1* were obtained with the SMART™ RACE cDNA Amplification Kit (Clontech, Mountain View, CA, USA). The primers *CaHsp70-1F* (5'-TCATCAACAAAATCTCTCTGT-3') and *CaHsp70-1R* (5'-GGAGCAAGAATAGACCACATA-3') were designed to clone the full-length cDNA of the pepper heat shock protein gene *CaHsp70-1*.

### 3.4. Sequence Analysis

The open reading frame (ORF) of *CaHsp70-1* was confirmed using the ORF Finder of NCBI [48] and the deduced amino acid sequence was analyzed using Compute *p*I/*M*_W_ tool [49] for computation of the theoretical iso-electric point and protein molecular weight. The prediction of the conserved domains, motifs and subcellular localization were carried out by InterProScan [50], PROSITE Scan [51] and WoLF PSORT [52], respectively. ClustalW program (Kyoto University Bioinformatics Center, Uji, Japan) and DNAMAN (Lynnon Biosoft, QC, Canada) were used for the sequence alignment and MEGA 5.1 software (Center for Evolutionary Medicine and Informatics of the Biodesign Institute, Tempe, AZ, USA) for constructing of the phylogenetic tree.

### 3.5. Subcellular Localization of CaHsp70-1

The ORF of *CaHsp70-1* without the termination codon was prepared by PCR using cDNA from R9 leaves treated with 40 °C for 2 h as the template and a primer set (forward primer 5'-GCTCTAGAATGGCGAAAGCTGAAGGCAAATC-3' and reverse primer 5'-CGGGGTACCATCCACTTCTTCAATTTTAGGGCCT-3'). The underlined nucleotides contain the *Xba*I and *Kpn*I restriction site, respectively. The PCR-amplified *CaHsp70-1* fragment was fused to the PBI221 expression vector. The CaMV35S::GFP (PBI221) vector without CaHsp70-1 was used as a control. For transient expression analysis, 5 µg of CaMV35S::CaHsp70-1-GFP plasmid or CaMV35S::GFP plasmid was introduced into the onion (*Allium cepa*) epidermal cells using a Bio-Rad He/1000 particle delivery system (Bio-Rad, Hercules, CA, USA). Bombarded cells were incubated for 24 h on 1× MS (Murashige and Skoog) agar medium, green fluorescent protein (GFP) fluorescence was observed using an A1R confocal-laser scanning microscope (Nikon, Tokyo, Japan).

### 3.6. Real-Time PCR Analysis

Real-time PCR for relative quantification of *CaHsp70-1* expression was performed in 20-μL reactions using 100 ng cDNA along with 0.8 µL of each 10 μM primer and 10 μL of SYBR Green Supermix (Takara, Dalian, China) in a Bio-Rad iCycler thermocycler (Bio-Rad IQ5TM). Ubiquitin binding protein gene *UBI-3* [53] was used as the reference gene. Nucleotide sequences of the gene-specific primers used were *CaHsp70-1* (forward primer 5'-TCAATGATTCTCAAAGGCAGGCTAC-3' and reverse primer 5'-GCAGTAGACTTCACTTCAAAAATCCC-3'), *UBI-3* (forward primer 5'-TGTCCATCTGCTCTCTGTTG-3' and reverse primer 5'-CACCCCAAGCACAATAAGAC-3'). The amplification protocol was 95 °C for 1 min, followed by 40 cycles of 95 °C for 10 s, 56 °C for 30 s and 72 °C for 30 s. The samples were performed in triplicate and three independent biological replicates were carried out.

### 3.7. The Promoter of CaHsp70-1 Analysis

The pepper genomic DNA was isolated from young leaves of B6 and R9 seedlings followed by NucleoSpin Tissue Genomic DNA Purification kit (Clontech). The promoter was cloned with the Universal GenomeWalker 2.0 kit (Clontech) according to the manufacturer’s instructions. *AP1* (5'-GTAATACGACTCACTATAGGGC-3') and *Hsp70-1–GSP1* (5'-GTGAAAGCAACGTAGGATGGAGTCGT-3') were used in the first amplification, *AP2* (5'-ACTATAGGGCACGCGTGGT-3') and *Hsp70-1–NGSP1* (5'-AACTGTAGGTCGTGCCGAGATCAATG-3') were used in the second amplification. Major bands (>1 kb) observed in secondary PCR were excised from the gel and purified using the NucleoSpin Gel and PCR Clean-Up kit (Clontech). The purified PCR products were cloned into pMD^®^19-T Vector (Takara) for sequencing. The promoters of *CaHsp70-1* analysis were performed by Plantcare [54].

### 3.8. Data Processing and Analysis

Expression of *CaHsp70-1* gene was analyzed with the Bio-Rad iQ5 (2.1 version) data analysis software; figures were done with Excel 2003.

### 3.9. Statistical Analysis

The statistical analysis of all data was performed by Statistical Analysis System software (version 8.2, SAS Institute, Cary, NC, USA) for one-way analysis of Variance (ANOVA) and the least significant range (LSR) method was used to compare the differences among means of treatments.

## 4. Conclusions

In summary, we cloned *CaHsp70-1* from thermotolerant line R9 seedlings under HS, and with the bioinformatics analysis, we confirmed that it belonged to the cytosolic Hsp70 subgroup. *CaHsp70-1* was positively responding to HS, and the expression patterns were different in various tissues, also in exogenous substances and HS treatments in different thermotolerant pepper lines. The function of *CaHsp70-1* gene in the formation mechanism of pepper thermotolerance still needs further research.
